# Assessment of dynamic material properties of intact rocks using seismic wave attenuation: an experimental study

**DOI:** 10.1098/rsos.170896

**Published:** 2017-10-11

**Authors:** W. A. M. Wanniarachchi, P. G. Ranjith, M. S. A. Perera, T. D. Rathnaweera, Q. Lyu, B. Mahanta

**Affiliations:** 1 Deep Earth Energy Laboratory, Department of Civil Engineering, Monash University, Building 60, Melbourne, Victoria 3800, Australia; 2 Department of Infrastructure Engineering, The University of Melbourne, Building 175, Melbourne, Australia; 3 School of Geosciences and Info-physics, Central South University, Changsha 410012, People's Republic of China; 4 Department of Earth Sciences, Indian Institute of Technology Bombay, Mumbai, India

**Keywords:** attenuation, *P* and *S* waves, dynamic mechanical properties, quality factor, attenuation coefficient

## Abstract

The mechanical properties of any substance are essential facts to understand its behaviour and make the maximum use of the particular substance. Rocks are indeed an important substance, as they are of significant use in the energy industry, specifically for fossil fuels and geothermal energy. Attenuation of seismic waves is a non-destructive technique to investigate mechanical properties of reservoir rocks under different conditions. The attenuation characteristics of five different rock types, siltstone, shale, Australian sandstone, Indian sandstone and granite, were investigated in the laboratory using ultrasonic and acoustic emission instruments in a frequency range of 0.1–1 MHz. The pulse transmission technique and spectral ratios were used to calculate the attenuation coefficient (*α*) and quality factor (*Q*) values for the five selected rock types for both primary (*P*) and secondary (*S*) waves, relative to the reference steel sample. For all the rock types, the attenuation coefficient was linearly proportional to the frequency of both the *P* and *S* waves. Interestingly, the attenuation coefficient of granite is more than 22% higher than that of siltstone, sandstone and shale for both *P* and *S* waves. The *P* and *S* wave velocities were calculated based on their recorded travel time, and these velocities were then used to calculate the dynamic mechanical properties including elastic modulus (*E*), bulk modulus (*K*), shear modulus (*µ*) and Poisson's ratio (*ν*). The *P* and *S* wave velocities for the selected rock types varied in the ranges of 2.43–4.61 km s^−1^ and 1.43–2.41 km h^−1^, respectively. Furthermore, it was observed that the *P* wave velocity was always greater than the *S* wave velocity, and this confirmed the first arrival of *P* waves to the sensor. According to the experimental results, the dynamic *E* value is generally higher than the static *E* value obtained by unconfined compressive strength tests.

## 1. Introduction

Understanding of the material properties of a substance is crucial to investigate the physical, chemical and thermal behaviours associated with it. Therefore, over the past decades, researchers have studied different types of materials to evaluate their usage in a broad range of applications. Among the different types of substances, rocks have a significant place, due to their applications in various disciplines including the energy sector, specifically in fossil fuels [1]. Considering energy and the environment, coal bed methane extraction [2], CO_2_ sequestration in deep geological formations [3], hydraulic fracturing of deep unconventional reservoirs [4,5] and deep geothermal energy [6] play important roles. All of the above-described processes are highly dependent on the material properties of the reservoir rock, which also varies highly with factors such as saturation medium, the degree of saturation, mineral composition of the rock and *in situ* stress conditions [7–9]. Well-bore drilling and hydraulic fracturing are two main processes associated with petroleum extraction and geothermal energy extraction [10], which are significantly affected by the strength properties of the rock [11]. In fact, it is a well-known fact that deep geological formations are formed according to different types of rock layers, and the material properties of these rocks are significantly different from each other. This makes fossil fuel exploration more difficult due to the fact that the drilling and fracturing processes need to consider all the existing rock materials. Therefore, fossil fuel and geothermal energy extraction are heavily dependent on the mechanical properties and saturation condition of the reservoir rocks, and precise knowledge of *in situ* mechanical properties and the saturation condition has become essential for effective fossil fuel and energy extraction. Therefore, researchers and scientists are increasingly investigating reservoir rock properties, specifically their strength and elastic properties, to make the fossil fuel and geothermal energy extraction more efficient and economical [7,12,13].

Various techniques are currently being used, and attenuation of seismic waves is a non-destructive technique [14] that can be used to investigate the mechanical properties of reservoir rocks as well as other substances [15]. Biwa [14] has studied the attenuation of waves in fibre-reinforced viscoelastic composites and found that when the wavelength is larger than the fibre radius, the attenuation coefficient is small compared with the viscoelastic matrix. Moreover, Pandit *et al*. [15] have characterized the wave attenuation in an elastic medium with voids and concluded that in elastic mediums with voids more than one wavefront might exist. Seismic waves can be used to investigate the mechanical properties of the reservoir rock as well as to identify the optimal drilling locations [16,17]. Chai *et al*. [17] confirmed that the rock joint properties including density and stiffness could be evaluated using seismic wave attenuation. Also, Jiang & Spikes [16] have developed a new procedure for seismic reservoir characterization and to locate optimum drilling locations. Body waves have a higher frequency and therefore travel faster than surface waves, and can be used to quantify the mechanical properties of rocks [18]. Body waves can be subdivided into two main types: *P* waves (primary or compressional waves) and *S* waves (secondary or shear waves). The attenuation of *P* and *S* waves in rocks depends on many factors, including the physical state and saturation condition of the rocks [19,20]. Under laboratory conditions, the attenuation characteristics of rocks can be measured using several techniques, including the resonant bar technique [21–23], amplitude decay of multiple reflections [24], slow stress–strain cycling [25] and pulse transmission [26–28].

To date, a number of studies have been conducted on the mechanical properties of different rocks under different saturation conditions using seismic waves. For example, Rathnaweera *et al*. [29] studied the attenuation of seismic waves in brine-saturated sandstone and found that attenuations in water and 10% NaCl saturated sandstone are similar. Moreover, according to Johnston *et al*. [30], properties including the number of cracks, distribution of cracks, type of pore fluid, fluid saturation and the mechanical properties of the rocks affect the attenuation of seismic waves in them. In addition, Johnston *et al*. [30] observed that in dry rocks the attenuation is less than that in saturated rocks. Even though most of the previous studies focused on *P* and *S* waves, Xia *et al*. [31] developed a new method to determine the quality factor for shear waves (*Q*_s_) using love waves. Bai [32] studied the correlation between the sonic velocity and rock density for different fields, and conducted a statistical analysis to evaluate the rock density using sonic velocity. Furthermore, many studies have been carried out on attenuation characteristics of other geo-materials such as sand and clay. As an example, Darendeli [33] has conducted a study on dynamic properties and behaviour of clay soils due to ground vibration and developed a set of empirical curves for seismic site response analysis. In addition, Payan *et al*. [34] studied the effect of particle shape on the damping ratio of soils due to free vibration and developed an expression for the damping ratio of soils subjected to confining stress.

However, because there have been very few studies related to the attenuation characteristics of dry rocks, understanding of the attenuation characteristics of different rock types remains limited. Therefore, a series of pulse transmission tests were conducted using acoustic emission (AE) and ultrasonic (UT) systems to investigate the attenuation characteristics of five different rock types: siltstone, shale, Australian sandstone, Indian sandstone and granite. The dynamic mechanical properties were also calculated, based on the seismic velocities (*P* and *S*), which can be used to identify the natural reservoir rock type prior to starting any fracturing or gas extraction process.

## 2. Experimental methodology

### 2.1. Sample preparation

For this study, five different types of samples were selected: siltstone, shale, Australian sandstone, Indian sandstone and granite. The selected samples cover two different continents, and the details of the geology of the samples are given in table 1. Rock samples were selected from five different basins in Australia, China and India. The geological maps of some of the basins are provided in the electronic supplementary material. It should be noted that all the rock samples were taken from outcrops and when selecting the samples, homogeneous samples were selected as much as possible to avoid the effect of existing layers.

**Table 1.** Selected samples with geographical locations.

rock type	location	image	details
siltstone	Eidsvold basin, Queensland Australia	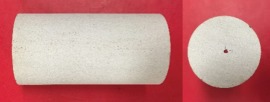	outcrop formed in the Triassic, Jurassic and Cretaceous periods almost homogeneous no visible layers
shale	Lower Cambrian Niutitang Formation, North-Western Hunan Province, China	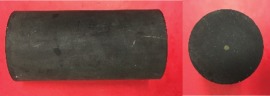	outcrop formed in the early Cambrian period organic rich black shale no visible layers
sandstone (Ind)	Dholpur, Rajasthan state, India	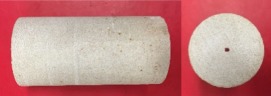	outcrop geology belongs to the Upper Bhander group medium-grained sandstone visible layers
sandstone (Aus)	Gosford basin, New South Wales, Australia	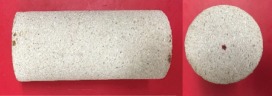	outcrop formed in the early Triassic period coarse-grained sandstone no visible layers
granite	Strathbogie batholith, Victoria, Australia	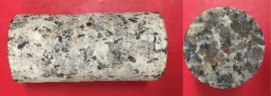	outcrop formation is a composite granitoid intrusion body coarse-grained highly discordant

Sample preparation and all the experiments were conducted in the Deep Earth Energy Research Laboratory of the Civil Engineering Department at Monash University. Samples were first cored into 38 mm cylindrical shapes and then cut into 76 mm lengths. Both end surfaces of the samples were carefully ground using a diamond grinding machine to produce parallel surfaces. Prepared samples were then oven-dried for 48 h at 35°C to remove the moisture. It should be noted that because the samples were taken from different basins, the natural moisture contents were different and this could affect the wave attenuation. Therefore, all the samples were oven-dried to remove the moisture. Prepared samples with their geographical origins are presented in table 1.

### 2.2. Testing procedure

**Figure 1.**
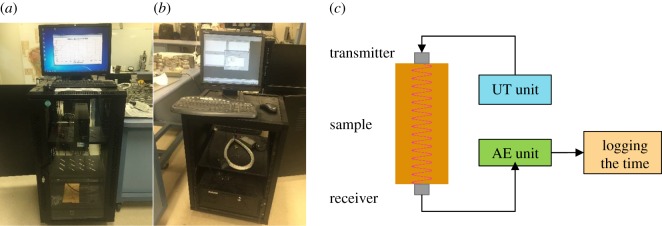
(*a*) Micro II UT system, (*b*) Micro II AE system and (*c*) mounting layout of the transmitter and the receiver.

Oven-dried samples were allowed to cool for 2 h prior to the experiment. Micro II AE and UT systems were used to receive and transmit the seismic pulses, respectively. Samples were located such that the central axis of the cylinder was along the vertical direction and the transmitter and receiver were attached to the top and bottom surfaces, respectively. It should be noted that before placing the transmitter and receiver, the end surfaces of the rocks were properly cleaned with a dry cloth and then white grease was applied to the sensor to act as an adhesive layer. Both the transmitter and receiver are cylindrical sensors 5 mm in diameter and 6 mm in height. The transmitter was directly connected to the Micro II UT system (figure 1*a*) and the receiver was directly connected to the Micro II AE system (figure 1*b*). The AE and UT systems were coupled together to record the transmitted signals directly through the AE system, and the mounting layout is shown in figure 1*c*. Experiments were carried out for a transmitting frequency range of 0.1–1 MHz (which has been identified as a suitable range for rock materials by Toksöz *et al*. [19] and Rathnaweera *et al*. [29]), and all the travel time values and amplitude values were logged on the data-logging software available in the AE system.

## 3. Results and discussion

### 3.1. Attenuation characteristics of different rocks

Attenuation of seismic waves can be described as the gradual loss in intensity of a wave when it travels through a medium [18,35]. Therefore, the best way to measure attenuation is based on the wave amplitude, and in this study the pulse transmission technique was used to measure the attenuation relative to a reference sample of steel (compared to rock, steel has very low attenuation, and it can be used as a reference to quantify the attenuation of different rocks). Therefore, all the experiments were conducted using identical procedures for all the rock samples as well as the steel sample to calculate the attenuation parameters. The amplitude of a plain seismic wave can be expressed as a function of frequency as follows [36]:

3.1 $$\begin{array}{r} A_{S}(\;f)=G_{S}(x)\;{\text{e}}^{-\alpha_{S}(\;f)x}{\text{e}}^{i(2\pi ft-k_{S}x)} \end{array}$$

and

3.2 $$\begin{array}{rl} A_{R}(\;f) & =G_{R}(x)\;{\text{e}}^{-\alpha_{R}(\;f)x}{\text{e}}^{i(2\pi ft-k_{R}x)}, \end{array}$$

where *A* is the amplitude, *f* is the frequency, *G*(*x*) is a geometrical factor, *x* is the distance between the sensor and the receiver (sample length), *k* is the wavenumber, which equals 2*πf*/*ν*, *ν* is the wave velocity, *α* is the attenuation coefficient, and subscripts S and R refer to the steel (reference) and rock sample, respectively. It should be noted that *G*(*x*) includes all the geometrical factors including spreading of the sensors, reflections, the mounting mechanism and sample size. Therefore, *G*(*x*) varies depending on the experimental conditions, and it is a unique value factor for a given sample under given test conditions. However, if the sample size and test conditions are the same for the reference and rock samples, then *G*(*x*) is the frequency-independent factor.

For the calculations, it is safe to assume that the attenuation coefficient (*α*) is a linear function of frequency within the frequency range of 0.1–1 MHz [37]. Then the attenuation coefficient can be expressed as the following equation:

3.3 α( f)=γ f,

where *γ* is a constant and can be calculated using the following equation [38]:

3.4 $$\gamma =\frac{\pi }{Q\;v}.$$

When the same sample size, transducers and mounting mechanism are used for both steel and rock, $G_{S}(x)/G_{R}(x)$ is frequency-independent, and equations (3.1) and (3.2) can be combined as equation (3.5) and then modified as equation (3.6):

3.5 $$\begin{array}{rl} \frac{A_{S}}{A_{R}} & =\frac{G_{S}}{G_{R}}\;{\text{e}}^{-(\gamma_{S}-\gamma_{R})fx} \end{array}$$

and

3.6 $$\begin{array}{r} \ln\left(\frac{A_{S}}{A_{R}}\right)=(\gamma_{R}-\gamma_{S})xf+\ln\left(\frac{G_{S}}{G_{R}}\right). \end{array}$$

**Figure 2.**
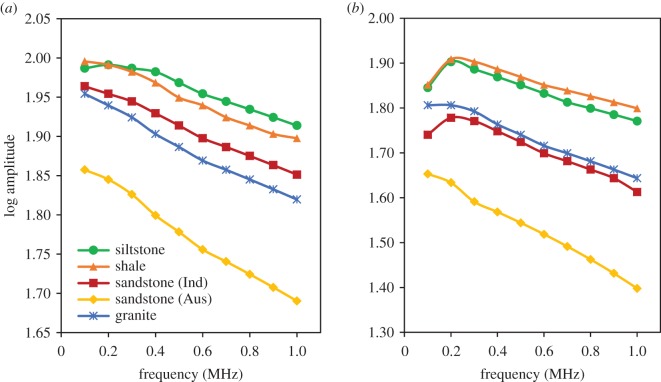
Variation of the log amplitude value with frequency for different rock types: (*a*) *P* waves and (*b*) *S* waves.

**Figure 3.**
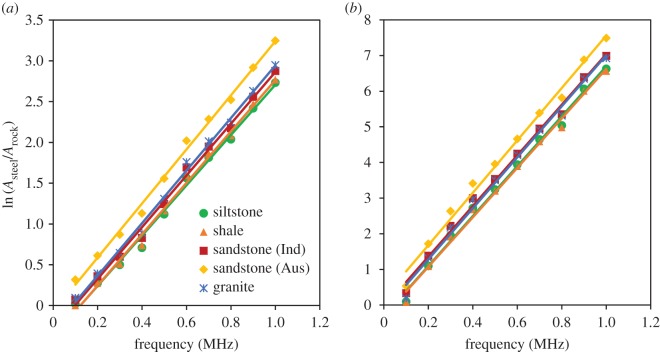
Attenuation characteristics of five different dry rocks with reference to the steel sample: (*a*) *P* waves and (*b*) *S* waves.

Based on equation (3.6), $\left(\gamma_{R}-\gamma_{S}\right)$ can be calculated for different rock samples by plotting the $\ln(A_{S}\text{/}A_{R})$ versus *f* graph. However, the *Q* value for steel is approximately 150 000 [39], which is approximately 4000 times that of the rocks. Therefore, based on equation (3.4), it is safe to assume $\gamma_{S}\approx 0$. Therefore, *γ*_s_ values were calculated by considering the gradient of the $\ln(A_{S}\text{/}A_{R})$ versus *f* graph and the sample length. The variations of the log amplitude with frequency for both *P* and *S* waves are shown in figure 2, and the variation of $\ln(A_{S}\text{/}A_{R})$ with frequency is shown in figure 3.

According to figure 2*a*, log amplitude values decrease with the increase in frequency of the *P* waves. This is because increased frequency causes higher energy loss when the *P* wave travels through the rock mass. However, according to figure 2*b*, the log amplitude values for *S* waves first increase with increase in frequency (up to 0.2 MHz) for Indian sandstones, siltstone and shale, and after that there is a gradual reduction in log amplitude values with increase in frequency. This suggests that the log amplitude values for *S* waves at 0.1 MHz are comparatively low (2.7% lower than the log amplitude values at 0.2 MHz). This may be due to the higher attenuation of *S* waves at 0.1 MHz, which causes an amplitude reduction. Interestingly, according to figure 2, Australian sandstone has the lowest log amplitude values for both *P* and *S* waves. Compared to the highest log amplitude values, Australian sandstone log amplitude values are approximately 11% and 22% lower at the 1 MHz frequency for *P* waves and *S* waves, respectively. This is possibly due to the higher porosity of the tested Australian sandstone, which is approximately 31% according to the results of mercury intrusion porosimetry tests. Seismic wave amplitudes drop significantly when they travel through a highly porous sample, due to the presence of air inside the sample.

Figure 3 shows the attenuation characteristics of *P* and *S* waves for the five selected different rock samples with reference to the steel sample with very low attenuation characteristics. Attenuation characteristics were interpreted as the natural logarithm of steel-to-rock amplitude ratios as a function of frequency. According to figure 3, it is clear that the $\ln(A_{S}\text{/}A_{R})$ value linearly increases with increase in frequency for all the five rock types, and Johnston & Toksöz [36] observed the same relationship for sandstone, limestone and shale samples. Using equation (3.6) and figure 3, *γ* values were calculated for all the rock types and both *P* and *S* waves. Interestingly, for *P* and *S* waves the *γ* value for granite is approximately 4.81 × 10^−5^ and 10.70 × 10^−5^ s m^−1^, respectively, which is approximately 20.3 and 18.8% higher than the values for the other rock types. This may be caused by the higher grain size of granite, which can dampen seismic waves when they propagate through the rock matrix [19]. This can be confirmed, as both the *P* and *S* waves show high *γ* values for granite.

**Figure 4.**
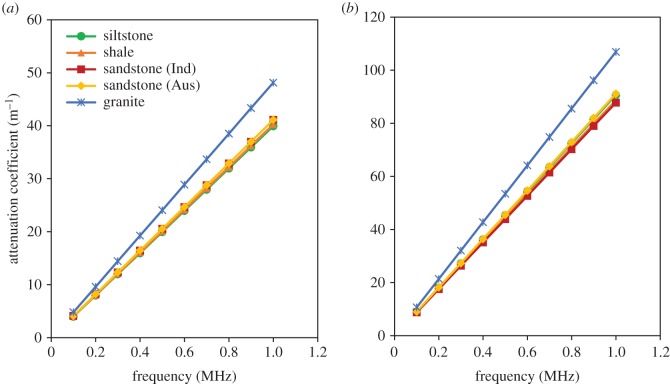
Variation of attenuation coefficient with frequency for different types of rocks: (*a*) *P* waves and (*b*) *S* waves.

Moreover, according to figure 4, granite has the highest attenuation coefficient values of 48.1 m^−1^ and 106.9 m^−1^ at the frequency of 1 MHz for *P* waves and *S* waves, respectively. However, for all other rock types, including sandstone, siltstone and shale, the attenuation coefficient is approximately 40 m^−1^ and 90 m^−1^ at the frequency of 1 MHz for *P* waves and *S* waves, respectively. Therefore, it is difficult to distinguish the rock types based on the attenuation coefficient. As a result, the quality factor (*Q*) values were calculated and the results are presented in table 2. As the table indicates, *Q* values were separately calculated for the *P* waves and *S* waves as *Q*_P_ and *Q*_S_, respectively. Interestingly, the *Q*_S_ values are always less than the *Q*_P_ values for all the rock types, and the same relationship has been observed by Rathnaweera *et al*. [29] for Hawkesbury sandstone. This can be explained using equation (3.4), where *Q* is inversely proportional to the product of seismic wave velocity (ν) and *γ*. Moreover, according to table 2, the highest *Q* value is observed for shale, and a similar value was observed by Johnston *et al*. [30]. Therefore, it is apparent that *Q*_P_ and *Q*_S_ can be used to identify the rock types.

**Table 2.** Calculated quality factor (*Q*) values for *P* waves and *S* waves.

	*Q*_P_		*Q*_S_	
rock type	mean value	standard deviation	mean value	standard deviation
siltstone	21.11	0.47	16.15	0.60
shale	32.16	0.36	24.72	0.73
sandstone (Ind)	25.77	0.40	19.63	3.29
sandstone (Aus)	28.17	0.72	23.08	1.22
granite	14.15	4.41	12.21	1.09

### 3.2. Dynamic mechanical properties of different rocks

The mechanical properties of a reservoir rock, including elastic modulus (*E*), bulk modulus (*K*), shear modulus (*µ*) and Poisson's ratio (*ν*), can be calculated as static or dynamic properties [40]. Static mechanical properties can be calculated from tri-axial or uni-axial tests (static tests), and dynamic mechanical properties can be calculated using seismic waves. In this study, dynamic mechanical properties were calculated using equations (3.7)–(3.10), based on the density, and *P* wave and *S* wave velocities [41–43]. The calculated dynamic mechanical properties are presented in table 3.

3.7 $$\begin{array}{rl} E & =\frac{\rho \;v_{S}^{2}(3v_{P}^{2}-4v_{s}^{2})}{v_{P}^{2}-v_{s}^{2}}, \end{array}$$

3.8 $$\begin{array}{rl} K & =\rho \;\left(v_{P}^{2}-\frac{4}{3}v_{s}^{2}\right), \end{array}$$

3.9 $$\begin{array}{rl} \mu & =\rho \;v_{s}^{2} \end{array}$$

3.10 $$\begin{array}{rl} \mathrm{and}\;\;\upsilon & =\frac{v_{P}^{2}-2v_{s}^{2}}{2(v_{P}^{2}-v_{s}^{2})}, \end{array}$$

where *E* is the dynamic elastic modulus, *K* is the dynamic bulk modulus, *µ* is the dynamic shear modulus, υ is the dynamic Poisson's ratio, *ρ* is the rock density, *ν*_P_ is the *P* wave velocity and *ν*_S_ is the S wave velocity.

**Table 3.** Seismic wave velocities and dynamic mechanical properties of different rocks (note that dynamic mechanical properties were calculated based on the mean velocity values).

	*V*_p_ (km s^−1^)		*V*_s_ (km s^−1^)						
rock type	mean value	standard deviation	mean value	standard deviation	*ρ* (kg m^−3^)	*E* (GPa)	*K* (GPa)	*µ* (GPa)	*ν*
siltstone	3.73	0.08	2.14	0.08	2239	25.80	17.35	10.30	0.25
shale	2.43	0.03	1.43	0.04	2661	13.45	8.39	5.46	0.23
sandstone (Ind)	3.11	0.05	1.82	0.25	2198	18.10	11.54	7.30	0.24
sandstone (Aus)	2.71	0.07	1.49	0.08	2202	12.59	9.65	4.91	0.28
granite	4.61	1.31	2.41	0.21	2616	39.78	35.42	15.15	0.31

According to table 3, the *ν*_P_ values are always higher than the *ν*_S_ values, because the travel time for *P* waves is less than that for *S* waves. Moreover, the obtained *P* and *S* wave velocities are within the range of 2–5 km s^−1^, which is consistent with the values obtained by Mavko [44]. Moreover, the *E* values and *υ* values are within the range of 10–40 GPa and 0.2–0.32 GPa, respectively, which is acceptable for rock materials [45]. Furthermore, the obtained values for *K* and *µ* are consistent with the values obtained by Toksöz *et al*. [41] for sandstone and granite. Therefore, it is evident that the *P* and *S* wave velocities can be used to calculate the dynamic mechanical properties of the five tested rock types. As can be seen in table 3, *P* and *S* wave velocities for Australian and Indian sandstones are quite different. This could be mainly due to the mineralogical contents of the two sandstones, with the quartz content approximately 85% and 95% in Australian and Indian sandstones, respectively. In addition, considering the grain size, Australian sandstone is a coarse-grained sandstone (grain size in the range of 0.04–1.0 mm), and the Indian sandstone is about fine- to medium-grained. Therefore, these differences may cause the significant variation between the *P* and *S* wave velocities in sandstones from the two continents. Table 4 gives a comparison of the dynamic and static elastic modulus and Poisson's ratio for the selected rock types. It should be noted that the static mechanical properties have been tested in the laboratory under previous studies, and further details can be found in Rathnaweera *et al*. [8], Sirdesai *et al*. [46] and Kumari *et al*. [47]. According to table 4, the dynamic elastic modulus is always greater than the static elastic modulus for all the tested rock types. This has also been observed by Yale [40] for dolostone, limestone, siltstone and mudstone. Moreover, the dynamic elastic modulus values of the siltstone, shale and sandstone are 22–78% higher than those of the static elastic modulus values, which is consistent with the range (15–70%) observed by Yale [40]. Furthermore, the ratio between the dynamic and static elastic modulus values is approximately 1.5. The higher dynamic elastic modulus values may be caused by geometric spreading, reflections, scattering and intrinsic damping, which can happen during wave propagation. However, according to table 4, granite has the highest dynamic elastic modulus, which is approximately 230% greater than the static elastic modulus. This may be due to the higher grain size of the granite, which may interfere in the propagation of seismic waves. In addition, granite is a crystalline rock with large grains (visible to naked eye), and this could enhance the wave propagation within the grain. However, all other rocks are sedimentary rocks. Moreover, because the grain boundaries are abundant within the rock itself, the seismic waves could easily reflect and interfere in the data logging. However, further experiments are required before a firm conclusion can be drawn.

**Table 4.** Comparison of dynamic and static elastic modulus (*E*_d_ and *E*_s_) and Poisson's ratio (*υ*_d_ and *υ*_s_) for different rocks (note that dynamic mechanical properties were calculated based on the mean velocity values).

		*E*_static_ (GPa)				ν_static_		
rock type	*E*_dynamic_ (GPa)	mean value	standard deviation	*E*_d_/*E*_s_	ν_dynamic_	mean value	standard deviation	*ν*_d_/*ν*_s_
siltstone	25.80	16.00	0.32	1.61	0.25	0.30	0.01	0.84
shale	13.45	7.56	0.14	1.78	0.23	0.21	0.02	1.11
sandstone (Ind)	18.10	11	0.21	1.65	0.24	0.31	0.01	0.77
sandstone (Aus)	12.59	10.29	0.68	1.22	0.28	0.26	0.02	1.09
granite	39.78	12.13	0.81	3.28	0.31	0.20	0.02	1.56

## 4. Conclusion

A laboratory experimental programme was conducted to investigate the attenuation characteristics of siltstone, shale, Australian sandstone, Indian sandstone and granite in an ultrasonic frequency range of 0.1–1 MHz. The following major conclusions can be drawn based on the results:

— The attenuation coefficient linearly increases with increase in frequency for all the tested dry rocks for both *P* and *S* waves.
— The logarithm of amplitude decreases with increase in frequency for all the tested dry rocks for both *P* and *S* waves.
— Compared to other rock types, the *γ* value and the attenuation coefficient of granite at all the frequencies are approximately 20.3 and 18.8% higher for *P* waves and *S* waves, respectively.
— In dry reservoir rocks, the *Q*_P_ value is greater than the *Q*_S_ value, and these values can be used to distinguish reservoir rocks.
— Reservoir acoustic logs can be used to determine the dynamic mechanical properties of rocks and to identify the rock types. Moreover, static mechanical properties can be estimated based on the dynamic mechanical properties (as an example, the ratio between the dynamic and static elastic modulus is approx. 1.5).

## Supplementary Material

Supplementary document

## Data accessibility

All data are included in the manuscript as tables and figures. Geological maps for the sample locations are separately included in the electronic supplementary material.
