# Supplementary material for: Assessment of dynamic material properties of intact rocks using seismic wave attenuation: an experimental study
Source: R Soc Open Sci. 2017 Oct 11;4(10):170896. doi: 10.1098/rsos.170896 (PMC5666273; doi:10.1098/rsos.170896)
Supplement: Supplementary document [file rsos170896supp1.docx]

**Supplementary document**

**Assessment of dynamic material properties of intact rocks using seismic wave attenuation: An ‎experimental study ‎**

**Journal:**

Royal Society Open Science

**Manuscript Title:**

Assessment of dynamic material properties of intact rocks using seismic wave attenuation: An experimental study

**Authors’ names:**

W.A.M. Wanniarachchi^1^, P.G. Ranjith^1*^, M.S.A. Perera^1, 2^, T.D. Rathnaweera^1^

^1^Deep Earth Energy Laboratory, Department of Civil Engineering, Monash University, Building 60, Melbourne, Victoria, 3800, Australia.

^2^Department of Infrastructure Engineering, The University of Melbourne, Building 175, Melbourne, Australia.

**Corresponding author:**

Prof Ranjith PG*

Deep Earth Energy Laboratory, Monash University, Building 60,

Melbourne, Victoria, 3800, Australia.

Phone/Fax: 61-3-9905 4982

E-mail: [ranjith.pg@monash.edu](mailto:ranjith.pg@monash.edu)

Geological map for the granite sample location


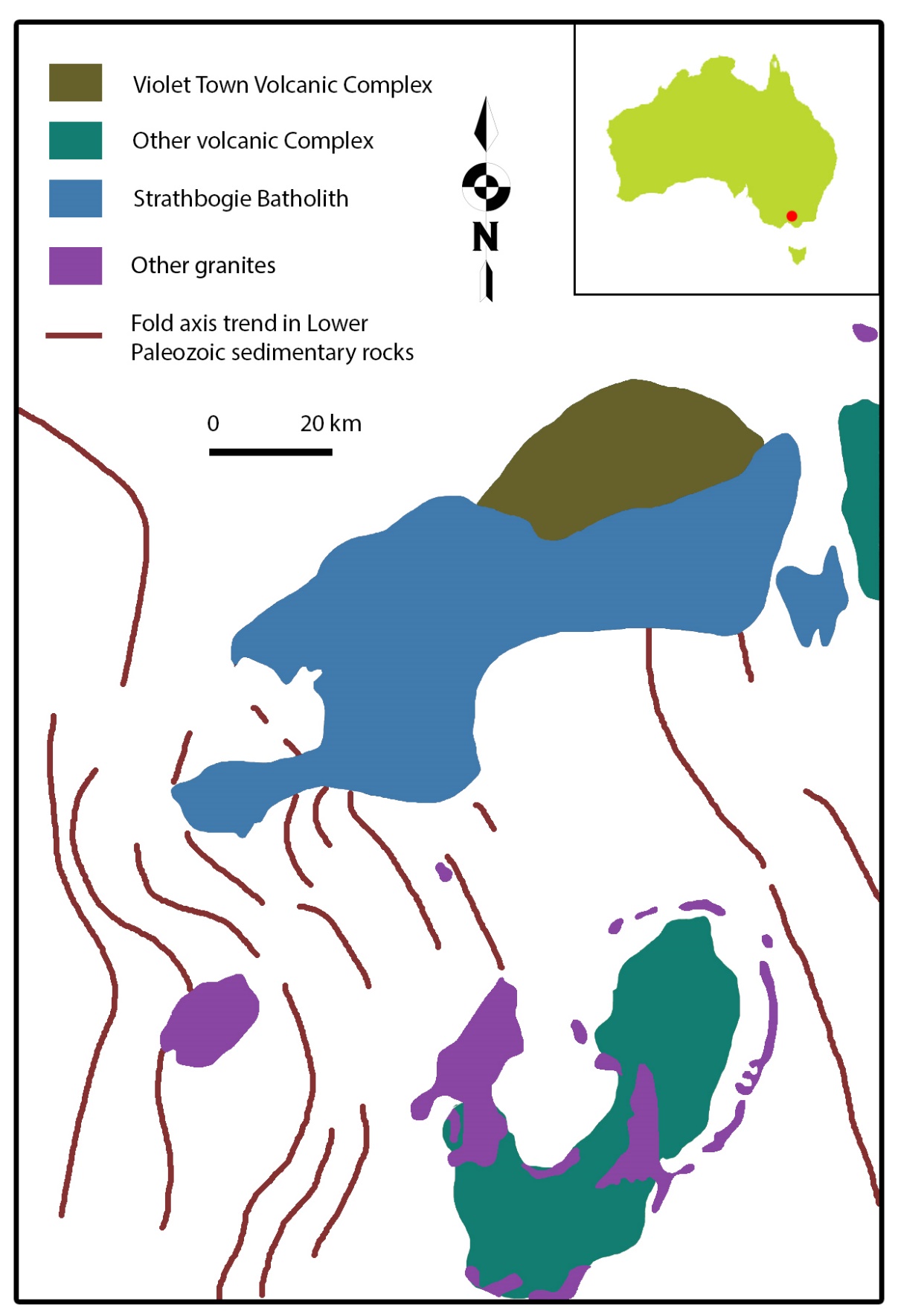


Supplementary figure 1: Geological map of the Strathbogie batholith (Modified after Phillips et al., 1981)

Geological map for the siltstone sample location


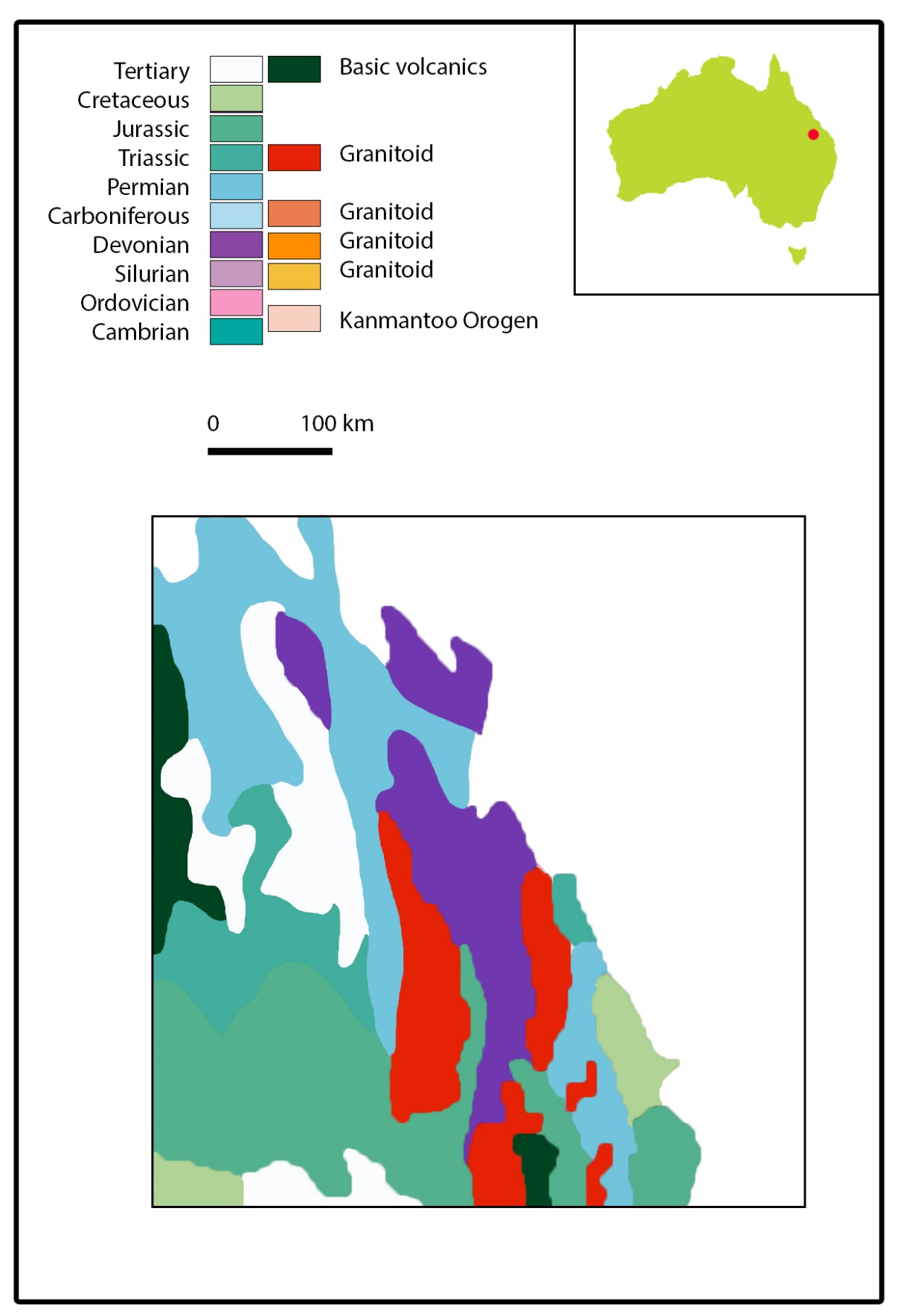


Supplementary figure 2: Geological map of the Eidsvold, Queensland

Geological map for the Australian sandstone sample location


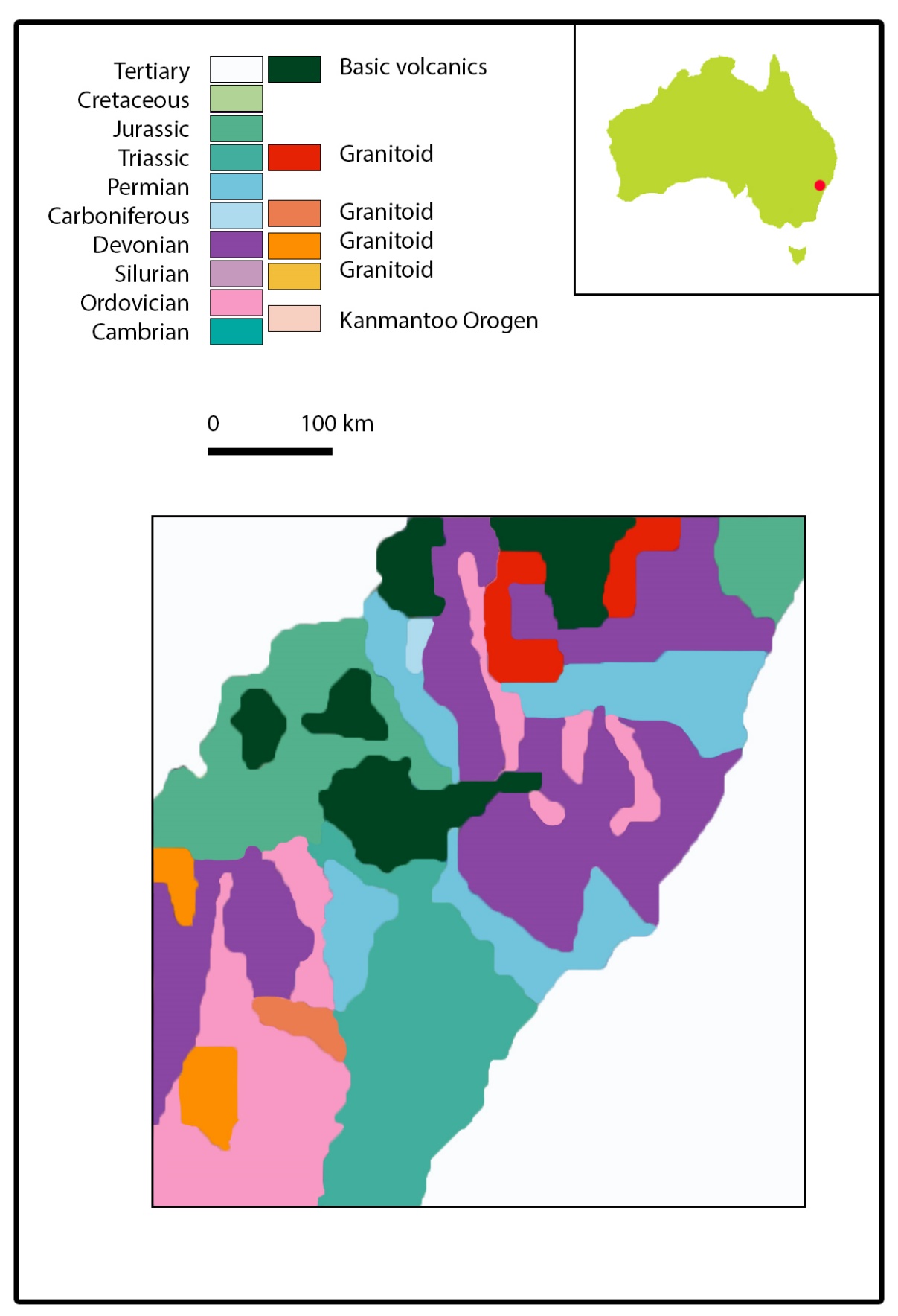


Supplementary figure 3: Geological map of the Gosford, Sydney

Geological map for the Indian sandstone sample location


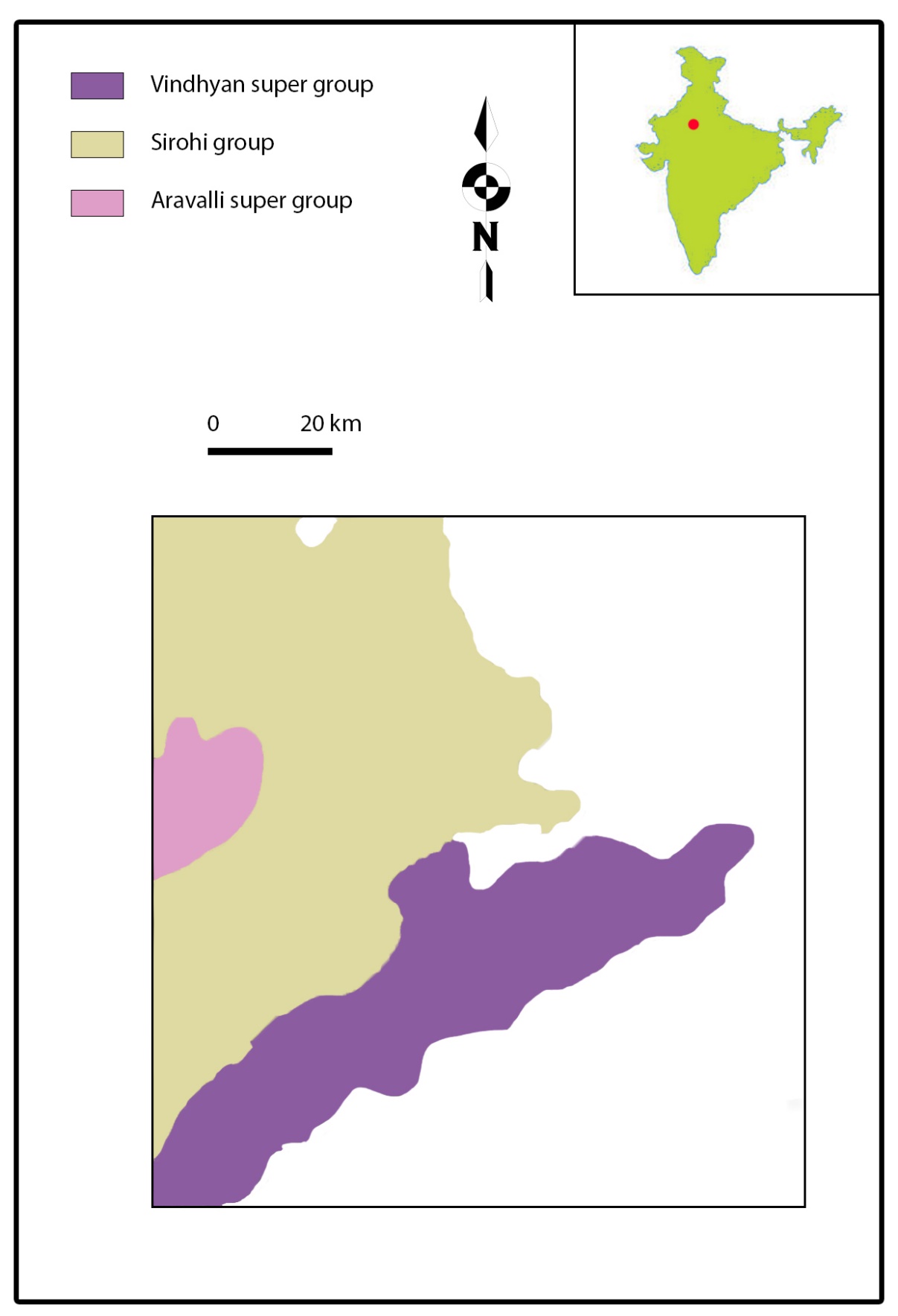


Supplementary figure 3: Geological map of the Dholpur, India
